# Artificial selection for resistance to copper and off-target physiological and behavioral effects in *Drosophila melanogaster*

**DOI:** 10.1016/j.ecoenv.2026.119974

**Published:** 2026-03-05

**Authors:** Kazzrie A. Arnold, Elizabeth R. Everman

**Affiliations:** School of Biological Sciences, University of Oklahoma, 730 Van Vleet Oval, Norman, OK 73019, USA

**Keywords:** Heavy metal resistance, Copper, Cross-tolerance, Artificial selection

## Abstract

Pollution resulting from mining, industry, and agriculture has played an important role in shaping the evolution of diverse organisms. Heavy metal contamination is of particular concern due to the health effects for humans and cross-tolerance effects that may influence pesticide resistance. We used a replicated artificial selection approach to examine the response to copper selection in *Drosophila melanogaster* collected from a retired mine and active fruit orchard. We tracked shifts in resistance to the target trait, adult copper resistance, as well as off-target effects on cadmium and lead resistance, starvation resistance, lifespan, and feeding aversion to copper contaminated food. Selection for copper resistance increased the focal trait and slowed the loss of resistance to non-essential heavy metals. Starvation resistance increased in response to copper selection, but was not sufficient to explain the increase in copper resistance. We also found that lifespan responded to copper selection in flies collected from one of the two collection sites, suggesting that life history traits may be influenced by repeated heavy metal exposure. Future genomic analysis will help clarify the genetic control of the selection response. Together, our results underscore the complexity of adaptive shifts in polygenic traits and provide a basis for further exploration of costs and correlative change following copper selection.

## 1. Introduction

Resistance to chemical stressors has repeatedly evolved in response to mining, agriculture, industry, and other anthropogenic sources in a wide range of organisms (Allen, 1971; Papadopulos et al., 2021; Diallo et al., 2022; Green et al., 2022; Cao et al., 2025). In some cases, the evolved response is accompanied by cross-tolerance (a correlated increase in resistance) to other environmental stressors (Augustyniak et al., 2017; Rodgers and Gomez Isaza, 2023; Bazzicalupo et al., 2025; Khan et al., 2025) and in other cases by changes in life history traits (Shirley and Sibly, 1999; Oliver and Brooke, 2018; Beribaka et al., 2021). Resistance to potentially toxic substances that are biologically necessary at low levels offers an opportunity to explore the process of complex trait adaptation and its potential consequences for other traits. The heavy metal copper is a conducive model stressor for this frame: copper is an essential micronutrient that plays a critical role in cellular respiration and the oxidative stress response (Slobodian et al., 2021), copper is toxic at high levels and can result in tissue and neurological damage (Stern, 2010), and resistance to copper toxicity is genetically complex (Everman et al., 2021), varies in natural populations, and is responsive to environmental exposure (Allen, 1971; Green et al., 2022; McNeilly, 1968; Lopes et al., 2004; Everman et al., 2023). Given the polygenic nature of copper resistance (Everman et al., 2023), previous links to variation in behavior (Everman et al., 2021), and resistance to other metals (Felmlee et al., 2022), selection for increased copper resistance has the potential to impact multiple traits.

The *Drosophila melanogaster* (fruit fly) model system has been used extensively to characterize the regulation, metabolism, and detoxification of copper and other heavy metals (e.g. Zn, Cd, Pb) (Felmlee et al., 2022; Calap-Quintana et al., 2017; Zhou et al., 2017; Rand et al., 2023). *D. melanogaster* has also been a powerful tool in artificial selection studies as it is often readily collected from the environment and can be maintained in large laboratory populations [e.g. (Novo et al., 2023; Lange et al., 2022). Because the genetic mechanisms underlying metal response are largely conserved between flies and other organisms (including humans) (Calap-Quintana et al., 2017; Fasae and Abolaji, 2022), studies that leverage *D. melanogaster* to characterize heavy metal resistance within an artificial selection framework have the opportunity to examine the effects of heavy metal selection on multiple potentially related traits.

In the present study, we examined the target and off-target effects of artificial selection for resistance to Copper(II) Sulfate in *D. melanogaster*. In previous work, we identified a high copper resistance population inhabiting a retired copper mine (Burra Burra Mine, BBM) in the Copper Basin of Tennessee, USA where mining occurred for six decades (Everman et al., 2023; Poste, 1932). Bulked-segregant analysis of flies from the BBM site revealed several candidate loci that have been previously linked to mitochondrial function, oxidative stress, arsenic and lead response, and ATP synthesis (Everman et al., 2023). The broad nature of the candidate gene categories raises the question of whether copper resistance can be further increased in the BBM population compared to another non-mine population, whether copper resistance influences resistance to other stressors, and whether there are implications for behavioral and life history traits as a result of evolved copper resistance.

Using flies collected from BBM and a nearby agricultural site in the Copper Basin region (Mercier Orchards (MO), Blue Ridge, GA, USA), we examined the effect of artificial selection for copper resistance on the target trait (adult copper resistance) as well as cadmium, lead, and starvation resistance using a replicated experimental evolution approach. We also tested the effect of copper selection on the behavioral trait feeding aversion to determine whether aversion to copper in food is responsive to copper selection. To investigate the potential effects of copper selection on life history, we measured variation in lifespan.

Overall, we found a clear positive effect of selection on adult copper resistance. In addition, loss of resistance to non-essential heavy metals lead and cadmium was slowed in flies from copper-selected cages. Control cages that were not subjected to selection lost cadmium and lead resistance over time. Consistent with patterns observed in wild populations (Everman et al., 2023), starvation resistance responded positively to selection for copper resistance, but resistance to copper was not fully explained by starvation resistance or increased aversion to copper-containing food. Instead, all flies (control and copper-selected) tended to lose their aversion to copper over time and the effect of selection was minimal. Finally, we observed an association between adult copper resistance and lifespan that varied depending on the source of the wild-derived flies. Flies from the BBM site retained longer lifespan regardless of selection, while MO-derived flies gained longevity in association with copper resistance. Future work will examine the genetic shifts through artificial selection to characterize potential pleiotropy between traits.

## 2. Materials and methods

### 2.1. Collections and rearing

Between July 09 and July 18, 2024, we collected gravid *Drosophila melanogaster* females daily from two locations (191 females from Burra Burra Mine, BBM (35.04 N, −84.38 W) and 355 females from Mercier Orchards, MO (34.89 N, −84.34 W)), which reside in or near the Copper Basin region of the United States (Figure S1A). Differences in the number of collected females may reflect differences in wild population size as sampling effort was similar between the two sites.

To collect flies, traps made from plastic bottles (ULINE, S-21727W) were prepared and baited with banana and yeast as described in Everman et al. (2023). Traps were suspended from structures (tree branches, railings) and were allowed to ferment for up to three days before replacement. In addition to passive trap collection, we used sweep netting to collect flies hovering near fruit sorting bins at the MO site and near dropped apples at the BBM site. We mouth-aspirated single female flies from traps or nets into cornmeal-molasses-yeast vials to collect eggs for species verification as described previously (Everman et al., 2023).

Generation 0 (G0) offspring of the species-verified *D. melanogaster* females were used to establish generation 1 (G1), from which five G1 males and females per original founder female were used to establish one population cage (30×30×30cm, Bugdorm-1) each for BBM- and MO-derived flies. Offspring were collected from all 191 or 355 founding females to establish each population-specific cage to maximize genetic diversity of the initial cages. Eggs were collected from G2 individuals in cages with apple juice plates baited with yeast paste following Everman et al (Everman et al., 2023)., resuspended in 10x PBS, and were pipetted into 24 6 oz *Drosophila* bottles (Genessee) containing media following the Bloomington *Drosophila* Stock Center Semi-Defined Food formulation (Backhaus et al., 1984). Bottles containing approximately 500 eggs each were used to establish six cages per collection site, resulting in a total of 12 wild-derived G3 cages (Figure S1B). All cages were maintained in the same Percival incubator at 25°C, 50% relative humidity, and under a 12:12 L:D cycle throughout the artificial selection process and for all phenotyping.

### 2.2. Baseline phenotyping of wild-derived flies

In addition to establishing the cages for MO and BBM-collected flies, we maintained the offspring of each founder female as isofemale strains in vials. Using G2 individuals, we measured Adult Copper Resistance (ACR) and Adult Starvation Resistance (ASR) to estimate the baseline levels of resistance in the MO and BBM collection sites in flies as close to field-caught as possible and prior to artificial selection (Figure S1B). Flies were sorted into groups of 20 individuals over CO_2_ and allowed to recover for 24 h on control food. Flies (3–5 days old) were then transferred to vials containing 1.8 g Instant *Drosophila* Media (Carolina Biological Supply Company 173200) hydrated with 8 mL 50 mM CuSO_4_ (Copper(II) Sulfate; Sigma-Aldrich C1297) or starvation media (1.5% agar, water, and phosphoric, propionic, and benzoic acid preservatives as described in as described previously (Everman et al., 2019). Survival was assessed every 24 h until all flies were dead. ACR and ASR were measured in 20 flies/sex/founder female/collection site.

### 2.3. Artificial selection and phenotyping of wild-derived population cages

Following the establishment of the replicate G3 population cages, we collected G4 eggs from each of the 12 cages (Figure S1B) into vials to obtain estimates for each of the traits that would be tracked through artificial selection. Unless otherwise noted, eggs were dispensed in aliquots of 30uL onto cornmeal-molasses-yeast media. Eggs were also collected into bottles (semi-defined media) to establish the next generation of cages (Figure S1B).

Selection for adult copper resistance occurred in every other generation, starting with G5 adults. Following emergence of adult flies in each cage, source bottles were removed and replaced with bottles containing semi-defined media with 50 mM CuSO_4_ in each copper-selected cage (4 bottles/cage). Control cages received four bottles of uncontaminated semi-defined media to prevent starvation. Once approximately 50% of flies had died in the copper-selected cages, we added another set of control bottles to all cages, including control cages to prevent starvation (8 bottles/selection cage, 4 bottles/control cage). We found that females typically laid fewer eggs after copper exposure, making apple juice plate egg collection in selection cages inefficient. Instead, we allowed females to lay eggs directly into the eight bottles to ensure a large enough population size in the next generation. In contrast, control cage females laid large numbers of eggs very quickly, necessitating collection of eggs with apple juice plates to control density.

Once egg collection was complete, adults from the selection generation were frozen and discarded to ensure non-overlapping generations. Cages were cleaned with warm water to remove all flies and bottles with even-numbered generation flies were placed into their respective cages. Once even-numbered generation flies emerged, we repeated the entire process through Generation 20 (8 selection events) as follows: We collected eggs into vials for phenotyping and collected eggs into bottles for the next generation, which were exposed to adult selection. We collected eggs from the generation following selection to decouple adult exposure from developmental effects on the offspring used for trait estimation. We estimate that census size of each population cage fluctuated between 1000 and 2500 flies in each generation, depending on whether the cages were experiencing selection or control conditions. Although we did not directly estimate effective population size in our study, previous work with evolving *D. melanogaster* populations suggests that effective population size may be lower than the census (Novo et al., 2023).

#### 2.3.1. Adult stress response

Flies used to assess stress resistance were reared on uncontaminated media, ensuring that no experimental individuals were directly exposed to copper prior to experiments. Three days following first emergence from vials, male and female flies were separated and sorted into groups of 20 individuals over light CO_2_ anesthesia and allowed to recover for 24 h. Following recovery, 3–5 day old flies were transferred to experimental vials containing 50 mM CuSO_4_ (ACR; 5 vials/sex/cage), starvation media (ASR; 5 vials/sex/cage), 20 mM CdCl_2_ (Cadmium Chloride Sigma 655198; ADR; 3 vials/sex/cage), or 100 mM Pb(CH_3_CO_2_)_2_ (Lead (II) Acetate Trihydrate, Sigma 228621; ALR; 3 vials/sex/cage). The lead and cadmium food were prepared in smaller quantities and with lower replication to reduce the impact of waste generated by our experiments in accordance with disposal guidelines at our institution. We used 1/8th of a teaspoon of finely ground Instant *Drosophila* media and hydrated food with 900uL of either metal in small plastic caps (MOCAP FCS131/16NA1) that fit within the narrow *Drosophila* vial opening. Caps were held onto vials with tape and disposed of following University guidelines. ACR and ASR vials were prepared as described above. Dead flies in each vial were counted daily to estimate survival and average lifespan (hours) per vial, per cage.

#### 2.3.2. Behavioral response to copper

We measured adult avoidance of copper contaminated food (CA) using the Microplate Feeding Assay developed by Walters et al (Walters et al., 2021) in both males and females (3–5 days old, 36 flies/sex/cage). This assay involved a 96-well plate containing 100uL of starvation media (prepared as described above) and a 3D printed coupler, which formed a connection between the starvation plate and a 1536-well plate containing 10uL liquid food dyed with 40ug/mL erioglaucine disodium salt (Sigma 861146) and different concentrations of copper. Flies were placed into the starvation plate (1 fly/well of rows 2–7 of each plate) and allowed to recover from CO_2_ and to fast for 24 h. Liquid food was prepared according to Walters et al (Walters et al., 2021) except that instead of ethanol, we tested 0 (control), 0.5, 1, and 2 mM CuSO_4_. Copper aversion at each concentration is referred to as CA0.5, CA1, and CA2 with respect to the concentration of CuSO_4_. The quantity of food consumed was estimated by comparing absorbance of food prior and following the feeding assay. We obtained initial absorbance readings at 630 nm for each 1536-well plate, allowed flies access to the food for 24 h, and then remeasured absorbance for each plate. After correcting for evaporation (Walters et al., 2021) and removing observations for flies that had died or not eaten food during the assay, we calculated preference per fly as (uL Copper Consumed – uL Control Consumed)/(Total uL Consumed). Because we calculated preference relative to the copper consumption, negative values indicate copper avoidance, and positive values indicate copper preference.

#### 2.3.3. Adult lifespan

Flies were collected for lifespan estimates (ALS) at the same time and in the same manner as for adult stress response traits. We measured adult lifespan in G4, G8, G12, G16, and G20 (3-day old starting age, 10 flies/5 vials/sex/cage). Flies were held on cornmeal-molasses-yeast media throughout the duration of their lifespan and were transferred to new vials on Monday, Wednesday, and Friday of each week. Counts of survival were made on each day that flies were transferred to new food. Average lifespan (days) was estimated for each vial per cage.

### 2.4. Data analysis

All data preparation and analyses were carried out in R (V 4.5.1) (R Core Team, 2023) and RStudio (V 2025.5.1.513) (Posit team, 2025). Significance was assessed at an alpha level of 0.05, and experiment-wide alpha levels were controlled for post hoc tests. Data visualization was accomplished using ggplot2 (Wickham, 2010). Unless stated otherwise, all analyses were implemented using functions from the stats R package (R Core Team, 2023).

#### 2.4.1. Baseline G2 assessment of ACR and ASR

After calculating average lifespan on either treatment per vial of 20 flies, data were analyzed with a two-way ANOVA (Average Lifespan ~ Sex * Collection Site) for each trait. Because we measured only one vial of females and males per founder female strain, the strains were treated as replicate estimates of each collection site. We also assessed the correlation between ACR and ASR, accounting for the effects of sex and collection site with multiple regression of all additive and interaction effects (ACR ~ Sex * Collection Site * ASR) using the lm function.

#### 2.4.2. Effect of selection and trait correlations

The focus of our experiment was to determine whether artificial selection for copper resistance would result in changes in average phenotype for each trait measured (ACR, ADR, ALR, ASR, ALS, CA0.5, CA1, and CA2) over time. To assess the influence of selection, we used analysis of covariance (ANCOVA) for each trait, which allows variation in the response to be partitioned according to categorical variables (sex, collection site, selection regime, cage, and treatment (feeding behavior)) while also testing the change in these variables against a continuous explanatory variable (generation). Because cage is confounded with collection site and selection regime, we accounted for cage as a model term but did not include cage in multi way interactions with collection site, selection regime, or treatment. Following initial assessment of ACR data, we found systematic bias in generation 6 (G6) data that was attributed to differences in experimenter handling of flies, so this generation was removed from analysis. As initial examination of data did not reveal obvious quadratic or other higher order patterns in the scatter of responses, we proceeded with linear models.

Often, ANCOVA is used to compare among treatment means at the grand mean of the covariate. Our usage of ANCOVA focused on the significance of the interaction terms, which provided a test of parallel slopes given that all cages began the artificial selection experiment with comparable levels of resistance, lifespan, and copper avoidance (Quinn and Keough, 2002). In the case that slopes were not parallel, this would indicate that the model term(s) leading to the significant interaction contributed to the divergence in phenotype over time. In particular, two-way interactions between selection regime and generation for each response were used to assess the statistical effect of selection. Three-way interactions allowed assessment of whether the change in response over time was due to different selection responses between the two populations (selection regime × collection site × generation), collection site-specific male and female responses (sex × collection site × generation), and whether the effect of selection was different between the sexes (sex × selection regime × generation). Four-way interactions (sex × selection regime × collection site × generation) for each trait were tested to determine whether the combined effects of the categorical model terms led to unique changes in the response over time. To further evaluate significant terms, post hoc tests were completed using estimated marginal means (emmeans_test) with Bonferroni correction using the R package rstatix (Kassambara, 2023).

The relative contribution of adult starvation resistance (ASR) to variation in every other trait was evaluated using forward stepwise linear regression using copper-selected cage-level averages for each trait, separated by sex. We initially determined that collection site did not significantly influence cage-level trait estimates, so we did not include collection site in the models (N = 6/generation/sex). In each model, we first estimated the adjusted R^2^ value for the reduced model (the effect of generation alone on ACR, ADR, ALR, ALS, CA0.5, CA1, and CA2) and then estimated the adjusted R^2^ value of the full model, where ASR was included in the models as an additive effect. The additive contribution of ASR to variation in each trait was determined by calculating the difference between the R^2^ values of the two models (full - reduced model) (Quinn and Keough, 2002).

Relationships between ACR (as the predictor variable) and the other metals (ADR and ALR as response variables) as well as the effects of copper avoidance at each concentration (CA0.5, CA1, and CA2 as predictor variables) on ACR were evaluated in the same manner as described above with individual forward stepwise regression models.

#### 2.4.3. Assessment of effect of selection versus G2 flies

We compared the adaptive response of MO and BBM flies to their respective G2 estimates of ACR using a three-way ANOVA, testing the effects of sex, generation (G2 vs G20), and collection site using data from the selection cages. Differences among G20 estimates of ACR between selection cages were determined with a three-way ANOVA (testing effects of sex, collection site, and generation) for G20 data followed by Tukey HSD post hoc comparisons.

### 2.5. Data availability

All phenotype data generated in this study are available from Fig-Share (10.6084/m9.figshare.29971417).

## 3. Results

### 3.1. Baseline ACR and ASR differed between collection sites

Baseline ACR (adult copper resistance) was variable in both collection sites, ranging from 51.6 to 120hrs in BBM females (48–88.8hrs in BBM males) and from 50.5 to 124.0hrs in MO females (44.6–86.4hrs in MO males). Flies derived from the BBM collection site had slightly higher ACR compared to MO flies (Collection Site: F_(1, 679)_ = 7.65, P < 0.006; Figure S2A; Table S1). The detection of significant differences between populations is likely influenced by large sample sizes and high power; therefore, it is possible that the difference in ACR between BBM and MO is not biologically meaningful. Males from both collection sites were consistently more sensitive to copper than females (Sex: F_(1, 679)_ = 553.29, P < 0.00001; Collection Site × Sex interaction: F_(1, 679)_ = 0.06, P = 0.8; Figure S2A).

Because flies in our study were exposed to copper in their food and flies may survive copper in part by avoiding consumption of contaminated food, we measured adult starvation resistance (ASR) and tested the relationship between ACR and ASR. ASR was similarly variable in each population (BBM females: 86.1–193.0hrs, BBM males: 68.6–141.0hrs; MO females: 97.2–182.0hrs, MO males: 74.7–140.0hrs) and BBM flies were slightly more starvation resistant compared to MO descended flies (F_(1, 591)_ = 4.69, P < 0.04; Figure S2B; Table S1). Females consistently survived much longer under starvation conditions compared to males (Sex: F_(1, 591)_ = 793.61, P < 0.00001; Collection Site × Sex interaction: F_(1, 591)_ = 1.03, P = 0.31; Figure S2B). ACR and ASR were significantly positively correlated (F_(7, 587)_ = 153, P < 0.00001, Adjusted R^2^ = 64%; Figure S2C). We also detected a small but significant interaction due to collection site, driven by a slightly stronger correlation between traits in MO flies (ASR × Collection Site: t = 2.49, P < 0.02; Figure S2C). Because starvation resistance has the potential to evolve in response to selection for copper resistance, we continued to track this trait throughout the artificial selection experiment.

### 3.2. Selection increased adult copper resistance

**Fig. 1.**
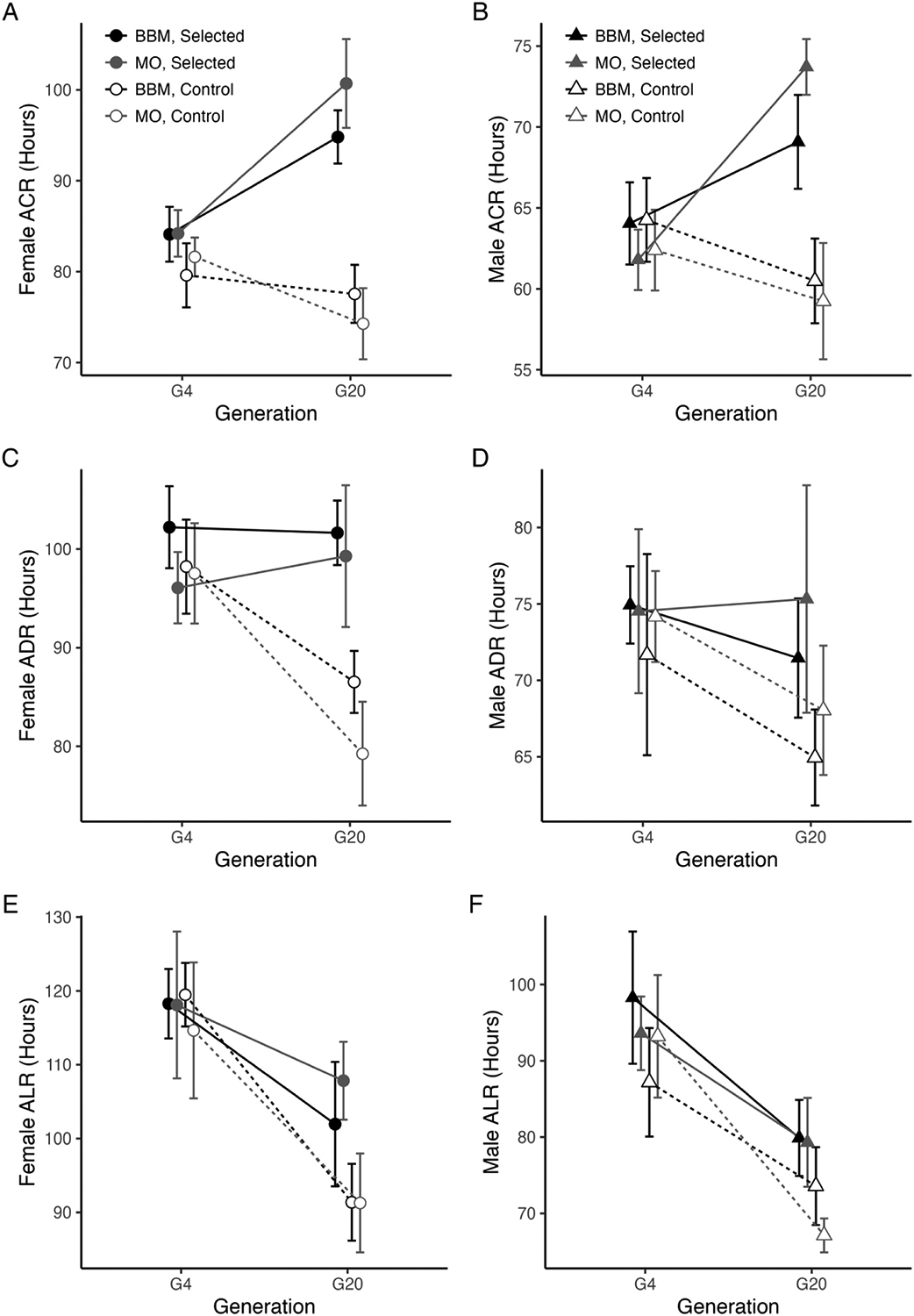
Selection for copper resistance influenced target and off-target metal resistance. A and B. Selection increased adult copper resistance (ACR) in adult flies in both BBM and MO-derived selected cages (P < 0.00001; Selection × Generation: P < 0.0001). Females (A) responded more strongly to selection compared to males (B; P < 0.004), and control cages generally retained baseline levels of ACR, despite a non-significant trend suggesting loss of ACR. C and D. Adult cadmium resistance (ADR) remained constant in response to selection and declined in control cages in females (C) and males (D) (Selection × Generation: P < 0.00001). E and F. Adult lead resistance (ALR) also did not increase with copper selection. Females (E) were more likely to retain ALR compared to males (F). In each panel, round symbols indicate females, triangles indicate males. Filled symbols and solid lines indicate copper-selected cages and open symbols and dashed lines indicate control cages. All data are presented as means across all vial replicates ± 95% confidence intervals. The legends provided in A and B apply to all following sex-specific plots. Statistics are presented in Table S2.

Copper selection significantly increased ACR relative to control (non-selected) cages, with copper-selected flies surviving on average 16.7 h longer than control flies on 50 mM CuSO_4_ by G20 (Selection: F_(1, 928)_ = 391.35, P < 0.00001; Selection × Generation: F_(1, 928)_ = 98.69, P < 0.00001; Fig. 1A and B; Table S2). The overall effect of selection was similar between BBM- and MO-derived flies (Collection Site: F_(1, 928)_ = 3.67, P = 0.06), and both BBM and MO cages responded to selection over time in a similar manner (Collection Site × Selection × Generation: F_(1, 928)_ = 0.0010, P = 0.97; Fig. 1A and B).

Both males and females responded to copper selection, though to different degrees (Sex × Selection: F_(1, 928)_ = 31.81, P < 0.00001). Overall, males had much lower resistance to copper stress (F_(1, 928)_ = 1992.26, P < 0.00001; Fig. 1B), and males displayed a weaker response to copper selection compared to females descended from either collection site (Selection × Sex × Generation: F_(1, 928)_ = 8.52, P < 0.004; Fig. 1B). Sex-specific differences in the response of ACR to selection are potentially influenced by the artificial selection process; adults were exposed until approximately 50% had died, but we expect that surviving females had previously mated to potentially less copper-resistant males. Surviving females may pass on less resistant alleles from prior mating with less resistant males, which could slow the response to selection overall or in a sex-specific manner if alleles have sex-specific effects. Alternatively, sex-specific differences in copper resistance may be due to physiological differences between males and females (such as body size and condition). Illumination of the mechanisms underlying sex-specific responses to copper requires further investigation. Post hoc comparisons of the first and last generations of selection showed that ACR remained essentially constant in the control cages in both sexes (BBM Females G4 vs G20: Bonferroni adj P = 1.0, MO Females G4 vs. G20: adj P = 0.07; BBM Males G4 vs. G20: adj P = 1.0; MO Males G4 vs. G20: adj P = 1.0; Fig. 1A and B).

### 3.3. Adult copper selection may influence resistance to lead and cadmium

Artificial selection for copper resistance has the potential to influence resistance to other heavy metals. Previous work has identified broad classes of oxidative stress, chemical stress response, and mitochondrial function genes that are associated with copper resistance in the BBM population (Everman et al., 2023). To examine the possible effect of copper selection on lead and cadmium resistance (non-target heavy metals), we measured resistance to these metals throughout the experiment. Overall, the effect of selection on ADR (adult cadmium resistance) was significant (Selection: F_(1, 616)_ = 70.39, P < 0.00001; Selection × Generation: F_(1, 616)_ = 30.12, P < 0.00001; Fig. 1C and D; Table S2). Interestingly, the effect of selection was driven by a loss of ADR in control cages rather than increased resistance in copper-selected cages. Female ADR was significantly higher compared to males (Sex: F_(1, 616)_ = 1010.28, P < 0.00001), and females from control cages lost ADR over the duration of the selection experiment (BBM Female G4 vs. G20: adj P < 0.007, MO Female G4 vs. G20: adj P < 0.00001; Fig. 1C). A similar non-significant trend was observed in males from both populations (Fig. 1D).

ALR (adult lead resistance) was also influenced by copper selection, with selection and control cages becoming more distinct over time (Selection: F_(1, 616)_ = 52.71, P < 0.00001; Selection × Generation: F_(1, 616)_ = 16.87, P < 0.00001; Fig. 1E and F; Table S2). Unlike ADR, flies from copper-selection cages were more likely to have a slight loss of ALR (BBM Female G4 vs. G20: adj P < 0.005, MO Female G4 vs. G20: adj P = 0.57; BBM Male G4 vs. G20: adj P < 0.0007, MO Male G4 vs. G20: adj P < 0.03). Similar to ADR, females from control cages derived from BBM lost resistance to lead most drastically over the course of the experiment, although the decline was also significant in MO-derived control females (BBM Female G4 vs. G20: adj P < 0.00001, MO Female G4 vs. G20: adj P < 0.00001; Fig. 1E). Males followed a similar trend with loss of ALR in control cages that reached significance for MO-derived control males (BBM Male G4 vs. G20: adj P = 0.05, MO Male G4 vs. G20: adj P < 0.00001; Fig. 1F).

ACR did not account for a substantial amount of variation in ADR or ALR after accounting for generation in either sex (Females ALR: t = 1.32, P = 0.19; Male ALR: t = −0.95, P = 0.35; Female ADR: t = −0.003, P = 1.0; Male ADR: t = −1.01, P = 0.32; Table S3). Taken together, our findings suggest that selection for copper resistance does not result in a correlated increase in lead or cadmium resistance at a phenotypic level that can be detected in our study. However, although power in our experiment is relatively low (N = 6 copper-selected cages measured at each generation), the effect of copper selection on non-target heavy metals warrants continued investigation, as copper selection slowed the decline of both ADR and ALR (Fig. 1C – F).

### 3.4. Starvation resistance increased in response to copper selection

**Fig. 2.**
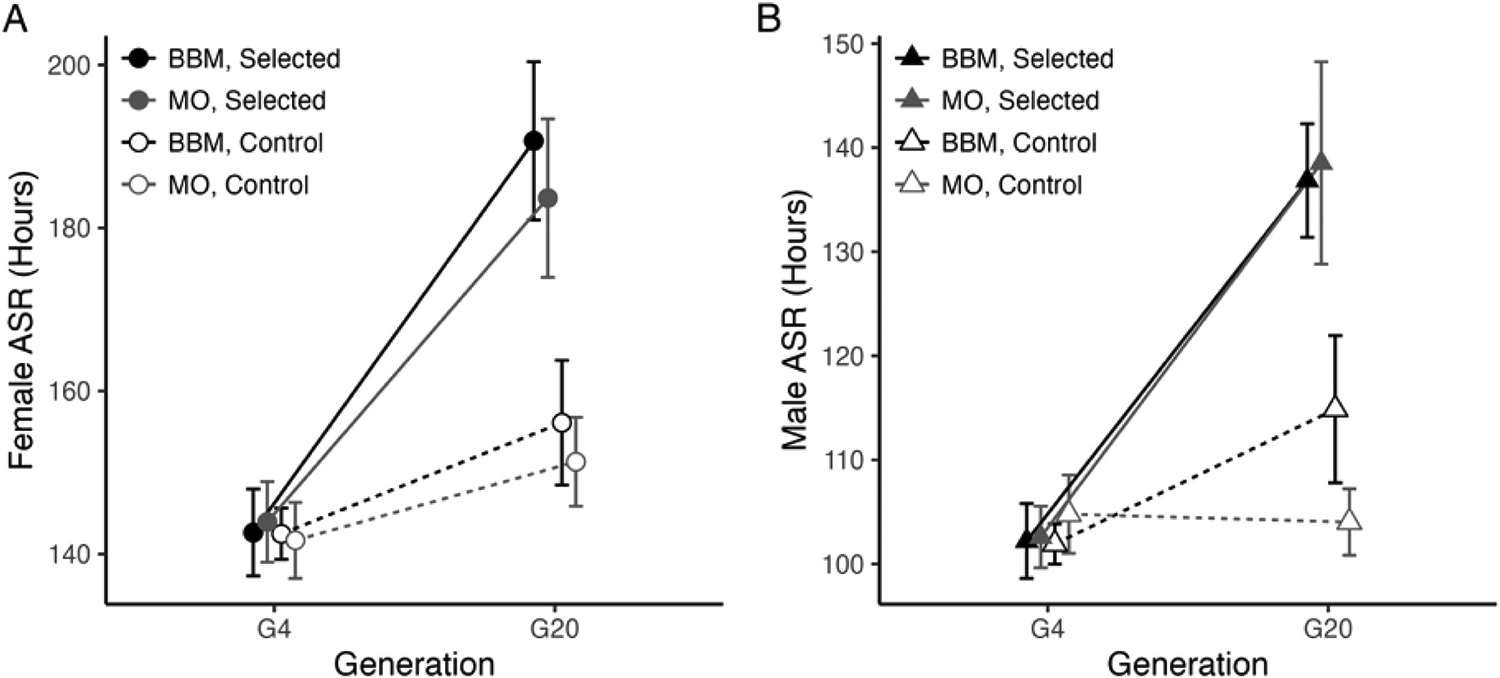
Adult starvation resistance (ASR) increased in response to copper selection. Females (A) and males (B) increased starvation resistance in copper-selected cages, while control flies remained largely constant. In A and B, data are presented as mean ± 95% confidence intervals. Round symbols indicate females, triangles indicate males. Filled symbols and solid lines indicate copper-selected cages and open symbols and dashed lines indicate control cages. Statistics are presented in Tables S4.

Selection for adult copper resistance had a strong positive effect on starvation resistance (ASR) (Selection: F_(1, 1047)_ = 278.23, P < 0.00001; Selection × Generation: F_(1, 1047)_ = 206.41, P < 0.00001; Fig. 2; Table S4). The response to selection was slightly stronger in BBM-derived cages compared to MO-derived cages (Collection Site × Generation: F_(1, 1047)_ = 26.02, P < 0.00001). ASR was significantly higher in females regardless of collection site (Sex: F_(1, 1047)_ = 3921.59, P < 0.00001; Sex × Population: F_(1, 1047)_ = 0.03, P = 0.87; Fig. 2A), and the response to selection was also greater in females compared to males (Sex × Selection: F_(1, 1047)_ = 22.66, P < 0.00001). ASR in control cages remained largely constant in both sexes with no significant decline (Fig. 2A and B).

**Fig. 3.**
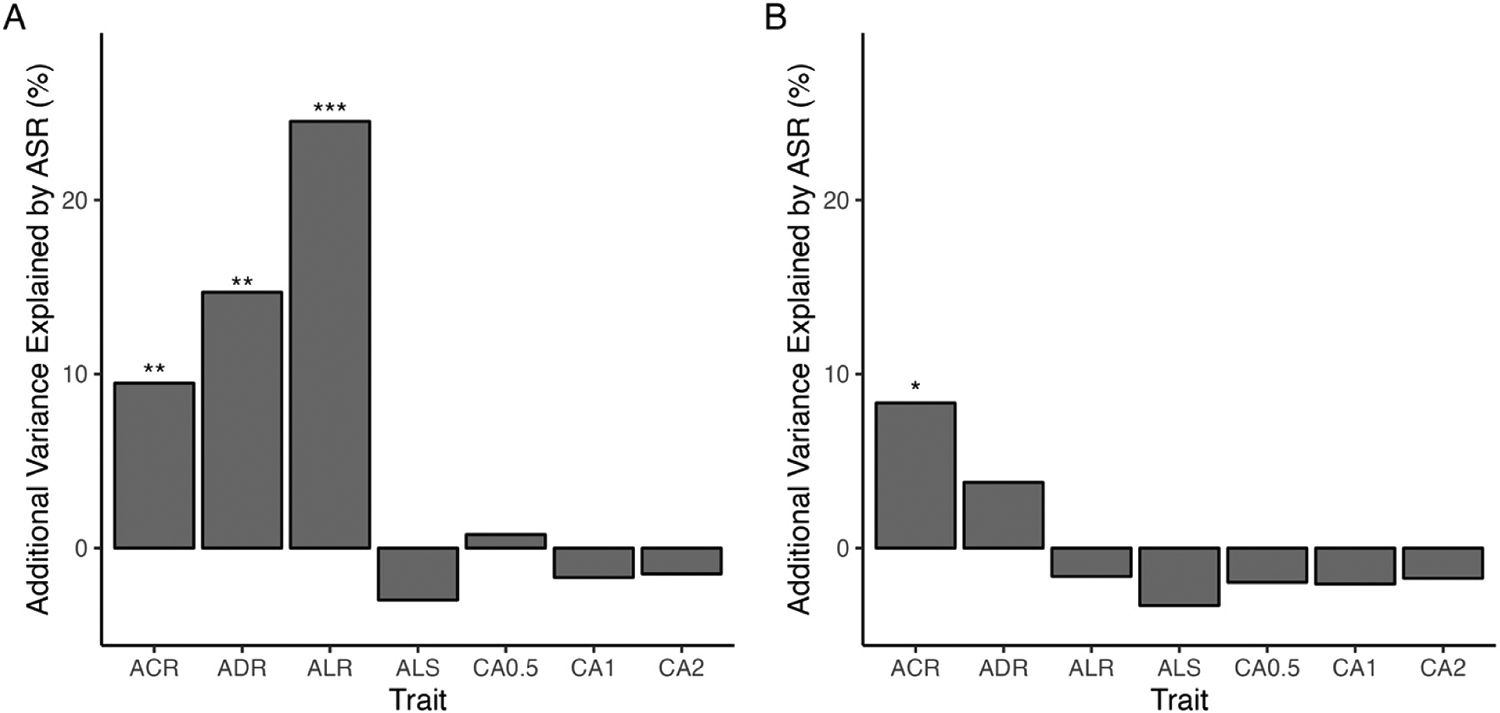
Additional variance explained by adult starvation resistance (ASR) following forward stepwise regression. A. Presents estimates for female flies from BBM and MO. B. Presents estimates for male flies from BBM and MO. ACR = Adult Copper resistance, ALR = Adult Lead Resistance, ADR = Adult Cadmium Resistance, ASR = Adult Starvation Resistance, ALS = Average Lifespan, CA0.5 = Copper Aversion to 0.5 mM CuSO_4_, CA1 = Copper Aversion to 1 mM CuSO_4_, CA2 = Copper Aversion to 2 mM CuSO_4_. Values are the additive contribution to the adjusted R^2^ estimate of models after accounting for the effect of generation. For example, in A, variation in ASR accounts for 10% of variation in ACR after accounting for the effect of generation. Asterisks indicate significance: * < 0.05, ** < 0.01, *** < 0.001. Collection site did not explain a significant proportion of variation for any trait, so was not included in the models. Estimates for each trait are based on the average cage-level response measured for copper-selection cages (N = 6 /generation/sex). Statistics are presented in Table S5.

After accounting for generation, ASR was significantly positively correlated with ACR in copper-selected cages in both males and females (Female ACR: t = 2.69, P < 0.01; Male ACR: t = 2.43, P < 0.05; Fig. 3; Table S5). While the correlations are significant and positive, the increase in ACR was not entirely due to an increase in ASR. After accounting for the effect of generation, ASR explained only 9.5% of variation in female ACR and 8.3% of variation in male ACR (Fig. 3).

We also assessed the contribution of variation in ASR to the non-target metal responses as these traits could be influenced by the capacity of flies to survive without consuming contaminated food. ASR explained a small but significant proportion of variation in ALR and ADR in females (ALR: 24.5% additional variation; ADR: 14.7% additional variation; Fig. 3A) but did not contribute to variation in non-target metal resistance in males (Fig. 3B). Given the unique and largely statistically independent responses of all resistance traits to copper selection (Figs. 1 and 2), our results suggest that copper selection may influence stress resistance as a more generalized and genetically diffuse trait.

### 3.5. Copper aversion was minimally affected by copper selection

**Fig. 4.**
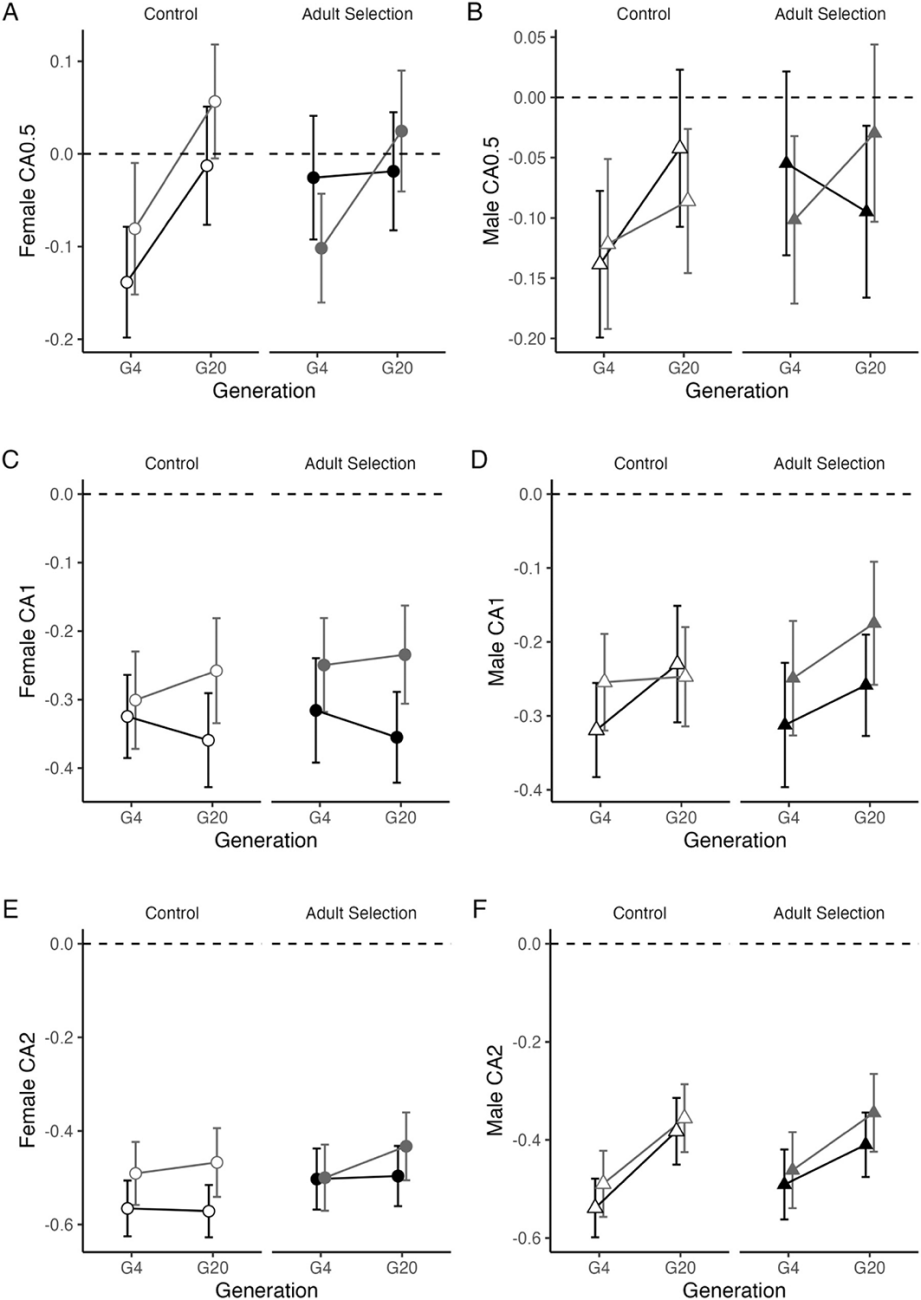
Copper selection had minimal effects on copper aversion (CA). A and B. Female and male flies tended to lose aversion to 0.5 mM over time. C and D. Female and male flies continued to avoid 1 mM copper food through the selection period. E. Females continued to avoid 2 mM copper food; F. We observed reduced aversion to 2 mM copper food in males over time. In all plots, round symbols indicate females, triangles indicate males. Open symbols indicate control cages; filled symbols indicate copper-selected cages. The dashed line at 0 indicates no preference between control and copper food. All negative values indicate aversion to copper. Data are presented as means ± 95% confidence intervals. Statistics are presented in Table S6.

In G4, male and female flies displayed aversion to even the lowest concentration of copper tested (CA0.5) (Fig. 4; Table S6), and the level of aversion was dose-dependent (Treatment: F_(2, 18536)_ = 1599.98, P < 0.00001). Overall, the effect of copper selection was minor (F_(1, 18536)_ = 8.16, P < 0.005, Table S6). With time, flies lost the strong aversion to low concentrations of copper (Treatment × Generation: F_(1, 18536)_ = 2.74, P = 0.06; Fig. 4A and B) but largely continued to avoid higher concentrations of copper (Fig. 4C – F). The loss of aversion was slightly more pronounced in control cages compared to copper-selection cages (Selection regime × Generation: F_(1, 18536)_ = 7.20, P < 0.008; Table S6). Aversion to copper at any concentration was not explained by ASR in either sex (Fig. 3; Table S5), nor did it explain variation in ACR (Table S7) suggesting that aversion to copper is not driven by copper resistance level or capacity for starvation resistance.

In general, our findings are consistent with previous studies that have reported copper aversion in *D. melanogaster* (Everman et al., 2021; Bahadorani and Hilliker, 2009). However, no studies to our knowledge have reported a loss of copper aversion over multiple generations in an artificial selection experiment. Loss of aversion to copper may be influenced by adaptation to lab conditions (Sgro and Partridge, 2001), especially if the effect of selection on copper aversion is not considered biologically meaningful in light of our high sample size and power to detect minor differences. In our experiment, copper avoidance measured through feeding behavior does not appear to be strongly linked to selection for increased copper resistance.

### 3.6. Selection for copper resistance increased lifespan

**Fig. 5.**
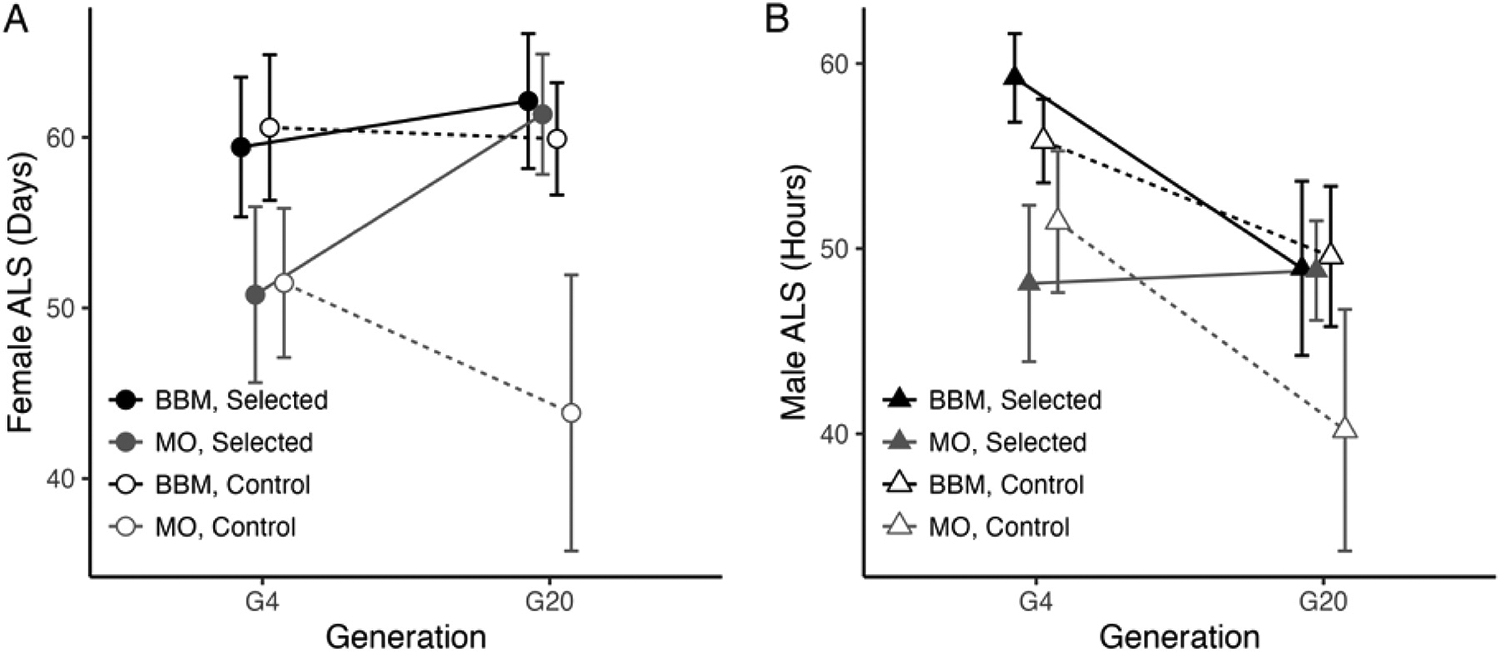
Adult copper selection had population- and sex-specific effects on longevity (ALS). A. MO-derived females from copper-selected cages gained longevity following selection, while BBM-derived female longevity remained constant in control and copper-selected cages. B. Males tended to lose longevity following selection in BBM-derived copper-selected cages and maintained longevity in MO-derived copper-selected cages. Data are presented as mean ± 95% confidence intervals. Round symbols indicate females, triangles indicate males. Filled symbols and solid lines indicate copper-selected cages and open symbols and dashed lines indicate control cages. Statistics are presented in Tables S8.

We found that selection for copper resistance influenced average lifespan in a collection site-dependent manner (Selection: F_(1, 592)_ = 105.29, P < 0.00001; Selection × Generation: F_(1, 592)_ = 22.25, P < 0.00001; Selection × Collection Site × Generation: F_(1, 592)_ = 13.93, P < 0.0003; Fig. 5; Table S8). BBM-derived cages either maintained (in females) or tended to lose longevity (males) in both copper-selected and control cages (Copper-selected cages G4 vs G20: Bonferroni adj P = 0.76; Control cages G4 vs G20: adj P = 1.0). In contrast, MO-derived flies from copper-selected cages gained on average 13.07 days of lifespan compared to MO-derived flies from control cages (Fig. 5).

Sex contributed to differences in average lifespan, with females living on average 6.12 days longer than males (Sex: F_(1, 592)_ = 106.32, P < 0.00001; Fig. 5). We detected a significant interaction between sex and generation (F_(1, 592)_ = 20.30, P < 0.00001) that was in part driven by a stronger response to selection in MO-derived females compared to MO-derived males (Copper-selected MO Females G4 vs G20: 10.6 day increase, Bonferroni adj P < 0.002; Copper-selected MO Males G4 vs G20: 0.70 day increase, adj P < 0.003). MO-derived males from control cages lost longevity (Control MO Males G4 vs G20: adj P < 0.0007). MO-derived control females followed the same non-significant trend (Control MO Females G4 vs G20: adj P = 0.06). In BBM-derived cages, females did not appear to respond to selection and maintained longevity in control cages (all G4 vs G20 adj P = 1.0). However, BBM-derived males tended to lose longevity in both copper-selected and control cages, although this trend only reached significance in males from copper-selected cages (Control G4 vs G20: adj P = 0.26; Copper-selected G4 vs G20: 10.30-day decrease, adj P < 0.003).

We found that ACR significantly contributed to increased lifespan in females, with ACR explaining an additional 10.5% of variation in ALS after accounting for generation. ACR did not significantly contribute to variation in male ALS from copper-selected cages(Table S3).

## 4. Discussion

### 4.1. Copper resistance increased in flies from two ecologically distinct collection sites

We collected flies from two sites chosen for their proximity to the Copper Basin, USA to explore the effects of artificial selection for copper resistance on target and off-target stress resistance, fitness, and behavioral traits. We previously demonstrated that copper resistance is high in the BBM population compared to other sites outside the Copper Basin despite the copper mine being retired for several decades (Everman et al., 2023; Poste, 1932). In contrast, MO, a large (27-acre) orchard, is potentially influenced by a combination of historical proximity to copper mines (being 16.84 km southeast of BBM) as well as contemporary routine use of organophosphate and Spinosad chemical control of pests (personal communication to ERE from MO owner).

Although our analysis of flies one generation removed from their field environment indicated statistical differences in ACR between MO and BBM, ACR was comparable between the two populations, suggesting the statistical difference may not be biologically meaningful (Figure S2). However, the adaptive responses of MO- and BBM-derived flies differed in our experiment. Comparison of flies as close to wild-caught as possible (G2) and G20 revealed that the increase in ACR was greater in MO males and females (16.48 hr increase) compared to BBM flies (9.1 hr increase) (F_(1, 739)_ = 7.79, P < 0.006). This difference in the overall adaptive response to copper selection may be due to differences in starting genetic diversity, evolutionary differences between the two populations, or a combination of these and other ecological and evolutionary factors.

The number of founding females differed between MO (355 females) and BBM (191 females) cages, and increased genetic diversity may have influenced the greater gain in ACR of MO-derived flies. The larger number of founders of the MO population cages increased the opportunity to include more rare alleles, which has the potential to influence the changes in genetic diversity in response to selection through our experiment. The differences in the starting levels of genetic diversity may become exacerbated over many generations of selection, because the effects of drastic reductions in population census on effective population size are expected to be more pronounced (Novo et al., 2023). We endeavored to avoid massive swings in population census by ending selection when approximately 1000 flies (primarily females) remained. Future analysis of genomic data will help clarify fluctuations in genetic diversity of each population cage through the experiment.

Prior exposure to insecticides may have also influenced the adaptive potential of MO-derived flies versus those collected from BBM. Correlations between resistance to heavy metals and insecticides have been previously reported in several species [reviewed in (Yan et al., 2024)] including mosquitoes (Oliver and Brooke, 2018; Poupardin et al., 2008), the beet armyworm (Augustyniak et al., 2017), and plant hoppers (Khan et al., 2025). Further, some pesticides contain copper as a means of control (Husak, 2015) and adaptive responses to copper-containing pesticides have been documented in fungi (Parry and Wood, 1958) and bacteria (Yu et al., 2022). Consistent with previous reports (Green et al., 2022; Everman et al., 2023), our sampling of flies from the active agricultural site suggests that insecticide use may be associated with increased resistance of *D. melanogaster* to copper toxicity as well. Alternatively, repeated exposure of the MO wild population to chemicals used in pest control may have influenced genetic predisposition or flexibility to respond to the copper stressor used in our study. Future examination of allelic variants underlying the shift in ACR between and among the MO and BBM cages will assist with determining whether, for example, broad oxidative stress response gene families are contributing to copper adaptation and whether the relatively consistent response to selection across different cage replicates is also repeatable at the genetic level.

### 4.2. Selection for copper resistance has the potential to influence resistance to other stressors

Selection for increased resistance to copper has the potential to result in correlated responses to other metals due to pleiotropic effects of genes involved in metal metabolism. A recent study in *Saccharomyces cerevisiae* examined the evolution of cross-tolerance to several heavy metals including copper and cadmium (Bazzicalupo et al., 2025). They found that cross-tolerance can evolve, but that it is difficult to predict and dependent upon the nature of the genetic changes that have led to increased resistance. Our examination of cross-tolerance between copper, cadmium, and lead revealed a similarly complex relationship at the phenotypic level. Copper selection did not result in a correlated increase in resistance to either non-essential metal (Fig. 1); however, cages subjected to copper selection retained resistance to cadmium and lost lead resistance at a slower rate. Cages maintained under control conditions significantly declined in cadmium and lead resistance over time, suggesting that there may be energetic costs associated with maintaining resistance to heavy metals and that copper selection has some influence over non-target metals.

In contrast to the lack of increased off-target metal resistance, we observed a collection site-specific, strong increase in starvation resistance in copper-selected cages (Fig. 2). An association between copper resistance and starvation resistance has been previously reported in *D. melanogaster* (Everman et al., 2023), and may be related to reductions in metabolic rate and activity levels as a result of selection (Everman et al., 2019; Rion and Kawecki, 2007). Response to metal toxicity is energetically taxing, and disruption to copper homeostasis can negatively impact the typical role of copper ions in mitochondrial function and metabolism (Ruiz et al., 2021). For example, in the ragworm (*Nereis diversicolor*), resistance to copper was linked to reduced growth and reproduction as well as reduced lipid and carbohydrate reserves (Pook et al., 2009). The increase in starvation resistance observed in both MO and BBM-derived copper-selected flies may be due adaptive responses to energetic stress.

Starvation resistance has also been linked to oxidative stress response at the genetic level (Tettweiler et al., 2005). We observed variation in the strength of the contribution of starvation resistance to copper, lead, and cadmium resistance (Fig. 3). If genes linked to shared stress responses (such as oxidative stress) are affected by selection, differences in correlation strength may be due to varying degrees of pleiotropy or quantitative effects of alleles among traits.

In general, behavioral resistance also has the potential to contribute to the mechanism underlying the link between copper and starvation resistance. In addition to altering physiological resistance to a selection pressure, exposure of insects to chemical stressors has been observed by others to increase aversion to a toxic chemical or the ability to modulate consumption to a level that is sublethal (Lockwood et al., 1984; Hubbard and Murillo, 2024). In our prior work using a large panel of inbred strains from the *Drosophila* Synthetic Population Resource (Everman et al., 2021), we found that strains with genotypes that were associated with low copper resistance tended to consume more copper-contaminated food, while strains with genotypes that were associated with high copper resistance tended to avoid copper contaminated food. These earlier observations illuminated a possible connection between the ability of flies to detect copper in their food and their resistance level. One explanation for this connection is that flies that avoid copper for a longer time ultimately increased their survival when exposed to copper by not consuming it. If selection for copper resistance results in increased aversion to copper, it is conceivable that both copper resistance and starvation resistance may increase because flies avoid eating copper-contaminated food for as long as possible. However, our study did not provide any evidence that selecting for increased adult copper resistance substantially influences the capacity of flies to detect copper in food or that it increases aversion of copper contaminated food (Fig. 4). In fact, flies from all cages (control and copper-selected) tended to either retain or lose aversion to copper over time (Fig. 4).

Loss of aversion at low copper concentration (CA0.5) may be due to lab adaptation, especially if aversion is dependent on continuous environmental cues or is costly to maintain (Sgro and Partridge, 2001). In natural populations or experiments with repeated exposures to a stimulus, habituation can also lead to a loss of aversion (Hall and Rodríguez, 2017). In these cases, repeated exposure can lead to less pronounced avoidance over time. In our study, all flies used in the feeding behavior assay were at least one generation removed from their cage environment and two generations removed from the most recent selection event. Flies used in the feeding assay were not exposed to copper until they encountered it in their food treatment, and they were exposed for the same amount of time (24 h). With these experimental parameters taken into account, habituation is unlikely. Overall, our assessment of feeding aversion to copper in flies subjected to selection for copper resistance suggests genetic independence between this behavior and physiological responses to copper selection in the populations examined in our study.

### 4.3. Fitness effects of copper selection are population specific

Negative consequences of metal exposure on survival and health have been extensively demonstrated in fish, arthropods, plants, and humans (Fasae and Abolaji, 2022; Pook et al., 2009), e.g (Weber et al., 2006; Gashkina, 2024). Less attention has been given to correlated shifts in fitness-relevant traits as a result of adaptation to metal stress. Our study offers a novel perspective on the consequences of adaptation to copper stress by tracking longevity throughout our selection experiment. We found that the effect of copper selection on lifespan was collection site-specific: BBM flies either maintained or tended to lose longevity independent of selection, while MO-derived flies gained longevity in tandem with copper resistance (Fig. 5). The mechanisms underlying this collection site-specific response require further investigation, but may be related to pleiotropic effects of genes that play a role in oxidative stress response. For example, in *Caenorhabditis elegans*, four genes that influence oxidative stress response with pleiotropic effects on metal resistance and longevity have been characterized (Barsyte et al., 2001; Tvermoes et al., 2010). Future analysis of the genetic basis of copper adaptation in the MO and BBM populations will allow us to investigate the potential for allele frequency shift at genes that influence copper resistance and lifespan.

In addition to possible genetic overlap, resource availability differs drastically between the BBM and MO Collection Sites and may contribute to site-specific changes in longevity. Large fruit crops are consistently available as a food source throughout the growing season at MO, whereas BBM lacks cultivated crops with the exception of a single apple tree. Natural selection for higher baseline lifespan in the BBM population may have resulted from more limited or more inconsistent opportunities for oviposition at this site. MO flies that are routinely exposed to insecticides may not be able to survive beyond early adulthood, potentially selecting against long life and favoring flies that reproduce early (Rose, 1984; Hubert et al., 2024). Prior work has established a strong connection between reproduction patterns and lifespan in *D. melanogaster*, with several classical quantitative genetic studies demonstrating a link between these two components of fitness [e.g (Rose, 1984; Rose et al., 1992; Hutchinson and Rose, 1991)]. We speculate that standing genetic variation in the MO population that overlaps with or is in linkage disequilibrium with variants associated with copper resistance may have increased in frequency during artificial selection, leading to increased lifespan. Our observations from the two collection sites that were the focus of this study provide justification for further exploration of the effects of habitat differences to determine if the population-specific patterns we observed are spurious (due to lab adaptation) or replicable across other agricultural and non-agricultural sites.

### 4.4. Conclusions

Understanding the consequences of adaptive responses from the perspective of cross-tolerance with other forms of stress can support a broader understanding for how genetically complex traits shift over time. We also gain a deeper understanding of the evolutionary potential of organisms to respond to ever-changing environments. Future genomic exploration and characterization of the traits examined in our study will additionally clarify the genetic response to selection. Together, our results underscore the complexity of adaptive shift in polygenic traits and provide a basis for further exploration of costs and correlative change following copper selection.

## Supplementary Material

1

2

## Data Availability

All phenotype data generated in this study are available from Fig-Share (10.6084/m9.figshare.29971417)

## Appendix A. Supporting information

Supplementary data associated with this article can be found in the online version at doi:10.1016/j.ecoenv.2026.119974.

## List of abbreviations

BBM: Burra Burra Mine
MO: Mercier Orchards
ACR: Adult Copper Resistance
ADR: Adult Cadmium Resistance
ALR: Adult Lead Resistance
ASR: Adult Starvation Resistance
ALS: Average Lifespan
CA(0.5,1,2): Copper Aversion at 0.5, 1, 2 mM CuSO_4_
