# Supplementary material for: Artificial selection for resistance to copper and off-target physiological and behavioral effects in Drosophila melanogaster
Source: Ecotoxicol Environ Saf. Author manuscript; Available in PMC 2026 Mar 24. (PMC13010382; doi:10.1016/j.ecoenv.2026.119974)
Supplement: 1 [file NIHMS2156246-supplement-1.docx]

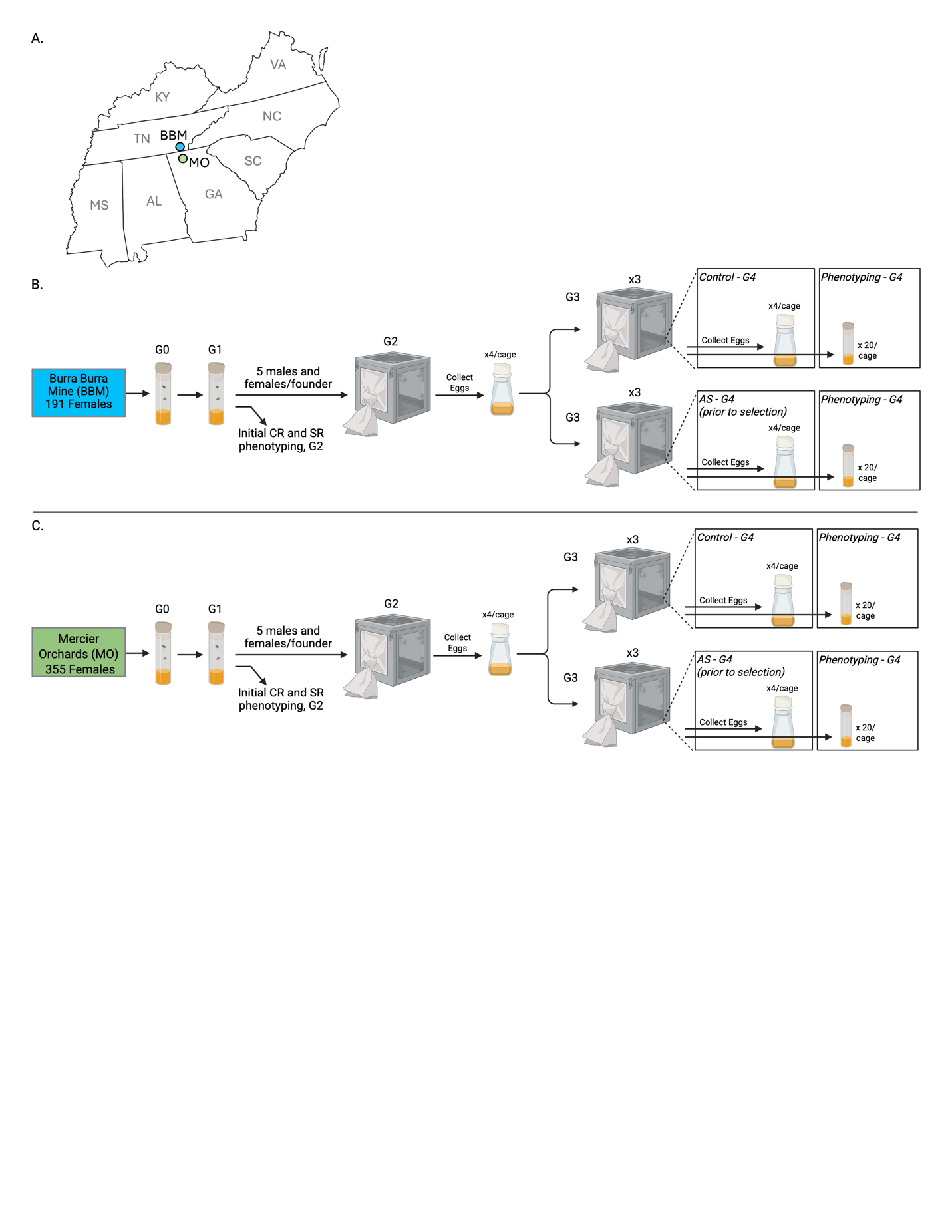


**Figure S1. Replicated cages were established from two wild populations of D. melanogaster.** A. Collection sites for Burra Burra Mine (BBM, blue) and Mercier Orchards (MO, green). B. We verified 191 gravid BBM females were D. melanogaster and used their offspring to establish one Generation 2 (G2) population cage and to measure baseline levels of copper resistance (CR) and starvation resistance (SR). After one generation of interbreeding, eggs were collected from G2 cages to establish three control and three adult selection (AS) G3 cages. Following a second generation of interbreeding, eggs were collected to establish G4 cages. We additionally collected eggs in vials to measure the following traits in G4 individuals: Adult Copper Resistance (ACR), Adult Lead Resistance (ALR), Adult Cadmium Resistance (ADR), Adult Starvation Resistance (ASR), Copper Aversion (CA), and Adult Lifespan (ALS). C. The same steps were followed to establish MO cages from the 355 species-verified founder females. This figure was created with BioRender.com.


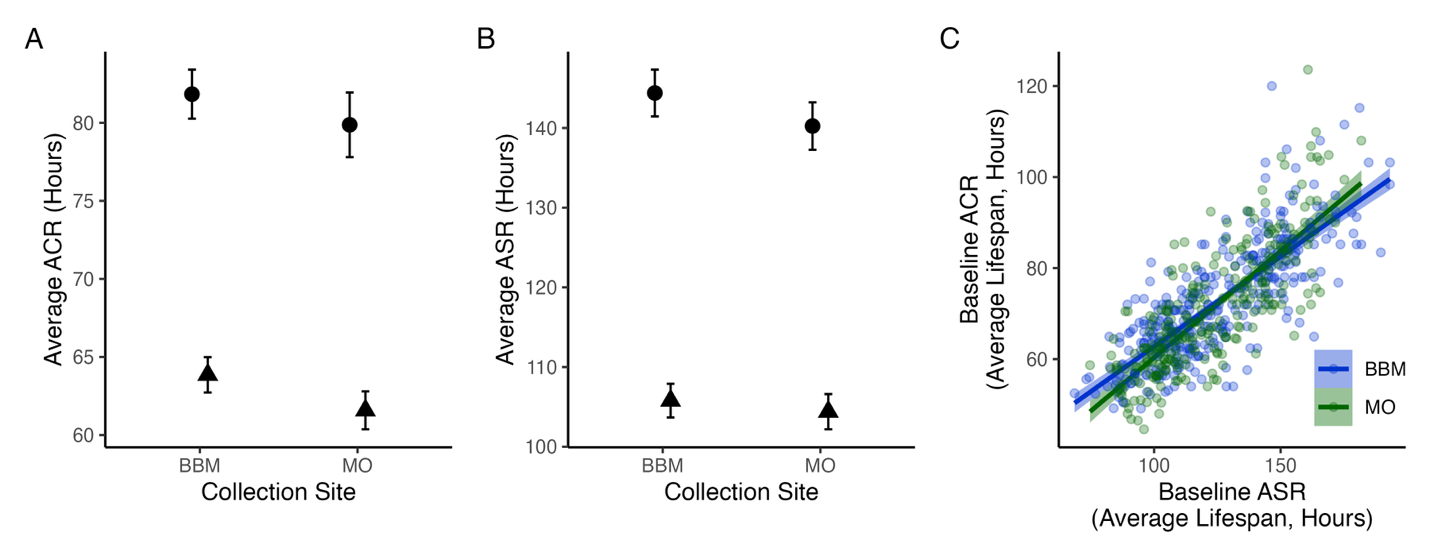


**Figure S2. Baseline ACR and ASR in BBM and MO descended G2 flies.** A. Baseline ACR (adult copper resistance) varied due to population and sex, with flies from BBM capable of surviving on 50mM CuSO_4_ slightly longer than MO flies (P < 0.006). Females consistently survived longer than males (P < 0.00001; Collection Site x Sex interaction: P = 0.81). B. Baseline ASR (adult starvation resistance) varied between BBM and MO (P < 0.04), and females were consistently more starvation resistant compared to males (P < 0.00001; Collection Site x Sex interaction: P = 0.31). C. ACR and ASR were significantly positively correlated (P < 0.00001, Adjusted R^2^ = 64%). The relationship between ACR and ASR was slightly stronger in MO compared to BBM (ASR x Collection Site: t = 2.49, P < 0.02). In A and B, data shown are means +/- 95% CI. In C, points indicate sex-specific isofemale strain means and the shading shows the 95% CI around the regression (solid line). Statistics are presented in Table S1.
