# Supplementary material for: Artificial selection for resistance to copper and off-target physiological and behavioral effects in Drosophila melanogaster
Source: Ecotoxicol Environ Saf. Author manuscript; Available in PMC 2026 Mar 24. (PMC13010382; doi:10.1016/j.ecoenv.2026.119974)
Supplement: 2 [file NIHMS2156246-supplement-2.docx]

# Supplemental Tables

Table S1. ANOVAs for ACR and ASR in G2 wild-derived flies.

| **Effect** | **DF** | **SS** | **MS** | **F Value** | **P Value** |
| --- | --- | --- | --- | --- | --- |
| **Adult Copper Resistance (ACR)** |  |  |  |  |  |
| Sex | 1 | 56198 | 56198 | 553.29 | < 0.00001 |
| Collection Site | 1 | 777 | 777 | 7.65 | < 0.006 |
| Sex x Collection Site | 1 | 6 | 6 | 0.06 | 0.81 |
| Residuals | 679 | 68966 | 102 |  |  |
| **Adult Starvation Resistance (ASR)** |  |  |  |  |  |
| Sex | 1 | 208490 | 208490 | 793.61 | < 0.00001 |
| Collection Site | 1 | 1232 | 1232 | 4.69 | < 0.04 |
| Sex x Collection Site | 1 | 271 | 271 | 1.03 | 0.31 |
| Residuals | 591 | 155263 | 263 |  |  |

Table S2. ANCOVA of the effect of selection on metal response.

| **Effect** | **DF** | **SS** | **MS** | **F Value** | **P Value** |
| --- | --- | --- | --- | --- | --- |
| **Adult Copper Resistance (ACR)** |  |  |  |  |  |
| Sex | 1 | 128807.57 | 128807.57 | 1992.26 | < 0.00001 |
| Selection | 1 | 25302.41 | 25302.41 | 391.35 | < 0.00001 |
| Collection Site | 1 | 237.17 | 237.17 | 3.67 | 0.06 |
| Cage | 9 | 2040.75 | 226.75 | 3.51 | < 0.0003 |
| Generation | 1 | 97.27 | 97.27 | 1.50 | 0.22 |
| Sex x Selection | 1 | 2056.59 | 2056.59 | 31.81 | < 0.00001 |
| Sex x Collection Site | 1 | 10.91 | 10.91 | 0.17 | 0.68 |
| Sex x Cage | 9 | 1043.02 | 115.89 | 1.79 | 0.07 |
| Collection Site x Generation | 1 | 1.11 | 1.11 | 0.02 | 0.90 |
| Selection x Generation | 1 | 6186.88 | 6186.88 | 95.69 | < 0.00001 |
| Sex x Generation | 1 | 9.17 | 9.17 | 0.14 | 0.71 |
| Selection x Collection Site x Generation | 1 | 0.07 | 0.07 | 0.00 | 0.97 |
| Sex x Collection Site x Generation | 1 | 11.92 | 11.92 | 0.18 | 0.67 |
| Sex x Selection x Generation | 1 | 551.15 | 551.15 | 8.52 | < 0.004 |
| Sex x Selection x Collection Site x Generation | 1 | 2.12 | 2.12 | 0.03 | 0.86 |
| Residuals | 928 | 59998.76 | 64.65 |  |  |
| **Adult Cadmium Resistance (ADR)** |  |  |  |  |  |
| Sex | 1 | 93047.97 | 93047.97 | 1010.28 | < 0.00001 |
| Selection | 1 | 6483.36 | 6483.36 | 70.39 | < 0.00001 |
| Collection Site | 1 | 964.50 | 964.50 | 10.47 | < 0.002 |
| Cage | 9 | 737.26 | 81.92 | 0.89 | 0.53 |
| Generation | 1 | 1916.94 | 1916.94 | 20.81 | < 0.00001 |
| Sex x Selection | 1 | 781.43 | 781.43 | 8.48 | < 0.004 |
| Sex x Collection Site | 1 | 68.33 | 68.33 | 0.74 | 0.39 |
| Sex x Cage | 9 | 1121.07 | 124.56 | 1.35 | 0.21 |
| Collection Site x Generation | 1 | 43.76 | 43.76 | 0.48 | 0.49 |
| Selection x Generation | 1 | 2773.80 | 2773.80 | 30.12 | < 0.00001 |
| Sex x Generation | 1 | 0.39 | 0.39 | 0.00 | 0.95 |
| Selection x Collection Site x Generation | 1 | 0.08 | 0.08 | 0.00 | 0.98 |
| Sex x Collection Site x Generation | 1 | 80.56 | 80.56 | 0.87 | 0.35 |
| Sex x Selection x Generation | 1 | 316.34 | 316.34 | 3.43 | 0.06 |
| Sex x Selection x Collection Site x Generation | 1 | 12.43 | 12.43 | 0.13 | 0.71 |
| Residuals | 616 | 56734.16 | 92.10 |  |  |
| **Adult Lead Resistance (ALR)** |  |  |  |  |  |
| Sex | 1 | 88365.55 | 88365.55 | 743.00 | < 0.00001 |
| Selection | 1 | 6268.77 | 6268.77 | 52.71 | < 0.00001 |
| Collection Site | 1 | 1462.00 | 1462.00 | 12.29 | < 0.0005 |
| Cage | 9 | 5734.26 | 637.14 | 5.36 | < 0.00001 |
| Generation | 1 | 18270.97 | 18270.97 | 153.63 | < 0.00001 |
| Sex x Selection | 1 | 0.39 | 0.39 | 0.00 | 0.95 |
| Sex x Collection Site | 1 | 96.93 | 96.93 | 0.82 | 0.37 |
| Sex x Cage | 9 | 981.79 | 109.09 | 0.92 | 0.51 |
| Collection Site x Generation | 1 | 13.28 | 13.28 | 0.11 | 0.74 |
| Selection x Generation | 1 | 2006.68 | 2006.68 | 16.87 | < 0.00005 |
| Sex x Generation | 1 | 1.84 | 1.84 | 0.02 | 0.90 |
| Selection x Collection Site x Generation | 1 | 197.76 | 197.76 | 1.66 | 0.20 |
| Sex x Collection Site x Generation | 1 | 264.39 | 264.39 | 2.22 | 0.14 |
| Sex x Selection x Generation | 1 | 329.99 | 329.99 | 2.77 | 0.10 |
| Sex x Selection x Collection Site x Generation | 1 | 251.03 | 251.03 | 2.11 | 0.15 |
| Residuals | 616 | 73261.54 | 118.93 |  |  |

Table S3. Forward stepwise regression testing the contribution of adult copper resistance to variation in ADR, ALR, ASR, and ALS.

| **Predictor** | **Response** | **Reduced Adj. R^2^** | **Full Adj. R^2^** | **Additive Variance Explained** | **P Value** |
| --- | --- | --- | --- | --- | --- |
| **Females** |  |  |  |  |  |
| ACR | ADR | 5.91% | 3.82% | -2.09% | 0.997 |
| ACR | ALR | -0.55% | 1.06% | 1.61% | 0.193 |
| ACR | ASR | 63.77% | 68.11% | 4.34% | 0.00989 |
| ACR | ALS | 18.18% | 28.71% | 10.53% | 0.0317 |
| ACR | CA0.5 | 7.22% | 8.99% | 1.77% | 0.1756 |
| ACR | CA1 | 1.89% | 2.32% | 0.42% | 0.2802 |
| ACR | CA2 | 4.78% | 2.75% | -2.04% | 0.849 |
| **Males** |  |  |  |  |  |
| ACR | ADR | 0.28% | 0.32% | 0.04% | 0.319 |
| ACR | ALR | 3.56% | 3.35% | -0.21% | 0.348 |
| ACR | ASR | 36.59% | 42.70% | 6.11% | 0.019146 |
| ACR | ALS | 1.36% | 1.53% | 0.17% | 0.315 |
| ACR | CA0.5 | 5.00% | 8.15% | 3.15% | 0.1154 |
| ACR | CA1 | 6.11% | 9.92% | 3.81% | 0.091 |
| ACR | CA2 | 11.38% | 12.56% | 1.18% | 0.20917 |

Table S4. ANCOVA of the effect of selection on ASR.

| **Effect** | **DF** | **SS** | **MS** | **F Value** | **P Value** |
| --- | --- | --- | --- | --- | --- |
| **Adult Starvation Resistance (ASR)** | | |  |  |  |
| Sex | 1 | 534387.79 | 534387.79 | 3921.59 | < 0.00001 |
| Selection | 1 | 37911.90 | 37911.90 | 278.22 | < 0.00001 |
| Collection Site | 1 | 24.92 | 24.92 | 0.18 | 0.67 |
| Cage | 9 | 9479.13 | 1053.24 | 7.73 | < 0.00001 |
| Generation | 1 | 37157.83 | 37157.83 | 272.68 | < 0.00001 |
| Sex x Selection | 1 | 3087.60 | 3087.60 | 22.66 | < 0.00001 |
| Sex x Collection Site | 1 | 3.82 | 3.82 | 0.03 | 0.87 |
| Sex x Cage | 9 | 387.67 | 43.07 | 0.32 | 0.97 |
| Selection x Generation | 1 | 28129.94 | 28129.94 | 206.43 | < 0.00001 |
| Collection Site x Generation | 1 | 3542.67 | 3542.67 | 26.00 | < 0.00001 |
| Sex x Generation | 1 | 2886.53 | 2886.53 | 21.18 | < 0.00001 |
| Selection x Collection Site x Generation | 1 | 302.40 | 302.40 | 2.22 | 0.14 |
| Sex x Selection x Generation | 1 | 362.82 | 362.82 | 2.66 | 0.10 |
| Sex x Collection Site x Generation | 1 | 0.17 | 0.17 | 0.00 | 0.97 |
| Sex x Selection x Collection Site x Generation | 1 | 527.17 | 527.17 | 3.87 | < 0.05 |
| Residuals | 1047 | 142672.72 | 136.27 |  |  |

Table S5. Forward stepwise regression testing the contribution of adult starvation resistance (ASR) to all traits.

| **Predictor** | **Response** | **Reduced Adj. R^2^** | **Full Adj. R^2^** | **Additive Variance Explained** | **P Value** |
| --- | --- | --- | --- | --- | --- |
| **Females** |  |  |  |  |  |
| ASR | ACR | 20.81% | 30.30% | 9.48% | 0.009891 |
| ASR | ADR | 5.91% | 20.62% | 14.70% | 0.00347 |
| ASR | ALR | -0.55% | 23.97% | 24.52% | 0.000249 |
| ASR | ALS | 18.18% | 15.20% | -2.98% | 0.903691 |
| ASR | CA0.5 | 7.22% | 8.00% | 0.77% | 0.245 |
| ASR | CA1 | 1.89% | 0.20% | -1.69% | 0.629 |
| ASR | CA2 | 4.78% | 3.30% | -1.49% | 0.591 |
| **Males** |  |  |  |  |  |
| ASR | ACR | 13.44% | 21.78% | 8.34% | 0.0191 |
| ASR | ADR | 0.28% | 4.06% | 3.78% | 0.101 |
| ASR | ALR | 3.56% | 1.93% | -1.63% | 0.63 |
| ASR | ALS | 1.36% | -1.95% | -3.31% | 0.765 |
| ASR | CA0.5 | 5.00% | 3.04% | -1.97% | 0.797 |
| ASR | CA1 | 6.11% | 4.05% | -2.06% | 0.917 |
| ASR | CA2 | 11.38% | 9.64% | -1.74% | 0.7365 |

Table S6. ANCOVA of the effect of selection on aversion to copper-contaminated food.

| **Effect** | **DF** | **SS** | **MS** | **F Value** | **P Value** |
| --- | --- | --- | --- | --- | --- |
| **Copper Aversion (CA)** | |  |  |  |  |
| Treatment | 2 | 451.28 | 225.64 | 1599.98 | < 0.00001 |
| Sex | 1 | 5.68 | 5.68 | 40.31 | < 0.00001 |
| Collection Site | 1 | 0.85 | 0.85 | 6.06 | < 0.02 |
| Selection | 1 | 1.15 | 1.15 | 8.16 | < 0.005 |
| Cage | 9 | 5.64 | 0.63 | 4.45 | < 0.00001 |
| Generation | 1 | 43.45 | 43.45 | 308.13 | < 0.00001 |
| Treatment x Sex | 2 | 12.42 | 6.21 | 44.02 | < 0.00001 |
| Treatment x Collection Site | 2 | 0.16 | 0.08 | 0.56 | 0.57 |
| Sex x Collection Site | 1 | 0.07 | 0.07 | 0.52 | 0.47 |
| Treatment x Selection | 2 | 0.70 | 0.35 | 2.49 | 0.08 |
| Sex x Selection | 1 | 0.54 | 0.54 | 3.80 | 0.05 |
| Treatment x Cage | 18 | 2.52 | 0.14 | 0.99 | 0.46 |
| Sex x Cage | 9 | 4.98 | 0.55 | 3.92 | < 0.00006 |
| Treatment x Generation | 2 | 0.77 | 0.39 | 2.74 | 0.06 |
| Collection Site x Generation | 1 | 0.04 | 0.04 | 0.31 | 0.58 |
| Selection x Generation | 1 | 1.02 | 1.02 | 7.20 | < 0.008 |
| Sex x Generation | 1 | 0.00 | 0.00 | 0.01 | 0.92 |
| Treatment x Sex x Collection Site | 2 | 0.47 | 0.23 | 1.66 | 0.19 |
| Treatment x Sex x Selection | 2 | 0.26 | 0.13 | 0.93 | 0.40 |
| Treatment x Sex x Cage | 18 | 1.14 | 0.06 | 0.45 | 0.98 |
| Treatment x Collection Site x Generation | 2 | 0.01 | 0.01 | 0.04 | 0.96 |
| Treatment x Selection x Generation | 2 | 0.02 | 0.01 | 0.09 | 0.92 |
| Collection Site x Selection x Generation | 1 | 0.00 | 0.00 | 0.02 | 0.88 |
| Treatment x Sex x Generation | 2 | 0.39 | 0.20 | 1.39 | 0.25 |
| Sex x Collection Site x Generation | 1 | 0.69 | 0.69 | 4.86 | < 0.03 |
| Sex x Selection x Generation | 1 | 0.14 | 0.14 | 0.97 | 0.33 |
| Treatment x Collection Site x Selection x Generation | 2 | 0.59 | 0.30 | 2.11 | 0.12 |
| Treatment x Sex x Collection Site x Generation | 2 | 0.06 | 0.03 | 0.23 | 0.79 |
| Treatment x Sex x Selection x Generation | 2 | 0.08 | 0.04 | 0.28 | 0.75 |
| Sex x Collection Site x Selection x Generation | 1 | 0.01 | 0.01 | 0.06 | 0.81 |
| Treatment x Sex x Collection Site x Selection x Generation | 2 | 0.08 | 0.04 | 0.27 | 0.76 |
| Residuals | 18536 | 2614.10 | 0.14 |  |  |

Table S7. Forward stepwise linear model testing the effect of copper avoidance (CA0.5, CA1, CA2) traits on ACR.

| **Predictor** | **Response** | **Reduced Adj. R^2^** | **Full Adj. R^2^** | **Additive Variance Explained** | **P Value** |
| --- | --- | --- | --- | --- | --- |
| **Females** |  |  |  |  |  |
| CA0.5 | ACR | 20.81% | 22.32% | 1.51% | 0.17564 |
| CA1 | ACR | 20.81% | 20.95% | 0.14% | 0.28016 |
| CA2 | ACR | 20.81% | 19.12% | -1.69% | 0.84897 |
| **Males** |  |  |  |  |  |
| CA0.5 | ACR | 13.44% | 16.31% | 2.87% | 0.1154 |
| CA1 | ACR | 13.44% | 16.95% | 3.51% | 0.0931 |
| CA2 | ACR | 13.44% | 14.60% | 1.16% | 0.2092 |

Table S8. ANCOVA of the effect of selection on average lifespan (ALS).

| **Effect** | **DF** | **SS** | **MS** | **F Value** | **P Value** |
| --- | --- | --- | --- | --- | --- |
| **Average Lifespan (ALS)** | |  |  |  |  |
| Sex | 1 | 5837.52 | 5837.52 | 106.32 | < 0.00001 |
| Selection | 1 | 5780.81 | 5780.81 | 105.29 | < 0.00001 |
| Collection Site | 1 | 6601.16 | 6601.16 | 120.23 | < 0.00001 |
| Cage | 9 | 10529.55 | 1169.95 | 21.31 | < 0.00001 |
| Generation | 1 | 364.30 | 364.30 | 6.64 | < 0.02 |
| Sex x Selection | 1 | 148.88 | 148.88 | 2.71 | 0.10 |
| Sex x Collection Site | 1 | 15.27 | 15.27 | 0.28 | 0.60 |
| Sex x Cage | 9 | 549.34 | 61.04 | 1.11 | 0.35 |
| Collection Site x Generation | 1 | 111.61 | 111.61 | 2.03 | 0.15 |
| Selection x Generation | 1 | 1221.74 | 1221.74 | 22.25 | < 0.00001 |
| Sex x Generation | 1 | 1114.39 | 1114.39 | 20.30 | < 0.00001 |
| Selection x Collection Site x Generation | 1 | 764.94 | 764.94 | 13.93 | < 0.0003 |
| Sex x Collection Site x Generation | 1 | 109.32 | 109.32 | 1.99 | 0.16 |
| Sex x Selection x Generation | 1 | 128.17 | 128.17 | 2.33 | 0.13 |
| Sex x Selection x Collection Site x Generation | 1 | 1.20 | 1.20 | 0.02 | 0.88 |
| Residuals | 592 | 32503.69 | 54.90 |  |  |
